# Synthesis of Silver-Strontium Titanate Hybrid Nanoparticles by Sol-Gel-Hydrothermal Method

**DOI:** 10.3390/nano5020386

**Published:** 2015-03-24

**Authors:** Shintaro Ueno, Kouichi Nakashima, Yasunao Sakamoto, Satoshi Wada

**Affiliations:** Graduate School Department of Interdisciplinary Research, University of Yamanashi, 4-4-37 Takeda, Kofu 400-8510, Japan; E-Mails: knakashima@yamanashi.ac.jp (K.N.); g14ma018@yamanashi.ac.jp (Y.S.); swada@yamanashi.ac.jp (S.W.)

**Keywords:** silver nanoparticles, strontium titanate, sol-gel-hydrothermal method, hybrid particles

## Abstract

Silver (Ag) nanoparticle-loaded strontium titanate (SrTiO_3_) nanoparticles were attempted to be synthesized by a sol-gel-hydrothermal method. We prepared the titanium oxide precursor gels incorporated with Ag^+^ and Sr^2+^ ions with various molar ratios, and they were successfully converted into the Ag-SrTiO_3_ hybrid nanoparticles by the hydrothermal treatment at 230 °C in strontium hydroxide aqueous solutions. The morphology of the SrTiO_3_ nanoparticles is dendritic in the presence and absence of Ag^+^ ions. The precursor gels, which act as the high reactive precursor, give rise to high nucleation and growth rates under the hydrothermal conditions, and the resultant diffusion-limited aggregation phenomena facilitate the dendritic growth of SrTiO_3_. From the field-emission transmission electron microscope observation of these Ag-SrTiO_3_ hybrid nanoparticles, the Ag nanoparticles with a size of a few tens of nanometers are distributed without severe agglomeration, owing to the competitive formation reactions of Ag and SrTiO_3_.

## 1. Introduction

The sol-gel method is well-known as one of the versatile methods to synthesize inorganic compounds, including metal oxides, complex oxides, chalcogenides, and so on. This method is a promising low-energy method; however, in the case of powder syntheses, a subsequent heating process is frequently needed to obtain the desired compounds, and agglomerated nanoparticles are usually obtained. On the other hand, the sol-gel method combined with a hydrothermal method, the sol-gel-hydrothermal method, is capable of synthesizing microstructure-controlled nanoparticles at a lower temperature. There are many reports on the synthesis of various kinds of functional oxides by the sol-gel-hydrothermal method [1,2]. Among them, we are especially interested in the synthesis of perovskite oxides with unique morphologies by the sol-gel-hydrothermal method, such as barium titanate (BaTiO_3_) nanoparticles [3], BaTiO_3_ hollow nanoparticles [4], BaTiO_3_ thin films [5], strontium titanate (SrTiO_3_) nanocubes [6] and sodium-potassium bismuth titanate ((Na_0.8_K_0.2_)_0.5_Bi_0.5_TiO_3_) nanowires [7]. These morphologies of the perovskite oxides synthesized by the sol-gel-hydrothermal method are frequently different from those obtained by the hydrothermal method [8,9].

We attempt to apply the sol-gel-hydrothermal method for a synthesis of metal-complex oxide hybrid materials, especially metal nanoparticle-loaded oxide materials, which are expected to be applied to multi-functional materials. For example, the various kinds of metal nanoparticle-loaded perovskite oxide hybrid particles were attempted to be applied to photocatalysts [10,11,12,13,14]. For the synthesis of such nanometer-scale hybrid particles, the sol-gel-hydrothermal method is considered to be appropriate, because relatively high-crystallinity materials can be obtained at low temperature and oxidization or the grain growth of metal particles can be suppressed. Moreover, the size and morphology of hybrid particles are able to be controlled by modifying the synthesis conditions.

In our previous report, we synthesized Ag-BaTiO_3_ hybrid particles by the sol-gel-hydrothermal method to fabricate Ag/BaTiO_3_ nanocomposite compacts for capacitor applications [15]. Such metal/insulator composite capacitors with distributed metal particles in the insulator layer have attracted much attention because of their extremely high capacitance over 10^4^ [16,17,18,19], and the distribution state of the metal particles is required to be precisely controlled, that is the metal particles should be insulated by the insulator layer and homogeneously distributed to enhance the dielectric breakdown strength. The metal-insulator hybrid particles are helpful for enhancing the distribution of the metal nanoparticles. The metal nanoparticles are fixed on the surface of the larger insulator particles, and thus, the aggregation of metal nanoparticles can be suppressed. Actually, by using the Ag-BaTiO_3_ hybrid particles as the component, the resistivity of the Ag/BaTiO_3_ nanocomposite materials was enhanced.

In this study, for the fabrication of the metal/paraelectric nanocomposite capacitors, Ag nanoparticle-loaded SrTiO_3_ hybrid particles were attempted to be synthesized from amorphous titanium oxide gels incorporated with Ag^+^ ions by the sol-gel-hydrothermal method. We prepared the precursor gels by mixing the Ag, Sr and Ti sources in various molar ratios, and the size and morphology of the synthesized hybrid particles were investigated.

## 2. Results and Discussion

### 2.1. Preparation of Precursor Gels

**Figure 1:**
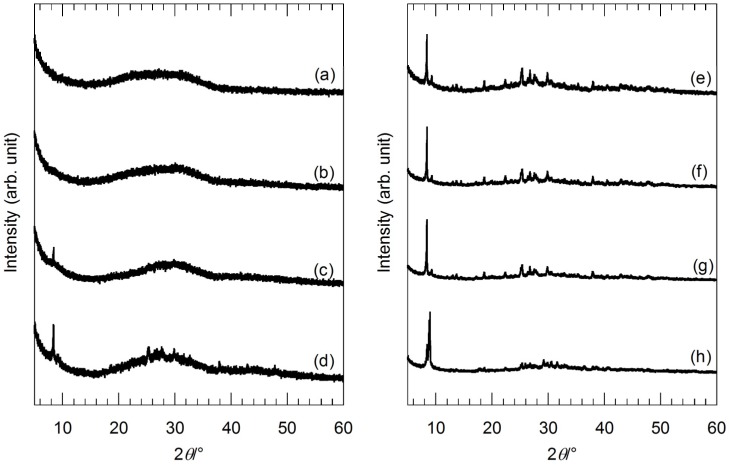
X-ray diffraction (XRD) patterns of the precursor gels with various molar ratios of (a) Ag:Sr:Ti = 1:0:4; (b) 1:1:4; (c) 1:2:4; (d) 1:3:4; (e) 0:4:4; (f) 1:4:4; (g) 2:4:4; and (h) 4:4:4.

Precursor gels were prepared by mixing and drying silver acetate (CH_3_COOAg) and strontium acetate hemihydrate (Sr(CH_3_COO)_2_·0.5H_2_O) mixed aqueous solution and titanium tetraisopropoxide (Ti[(CH_3_)_2_CHO]_4_) ethanolic solution. Figure 1 shows the X-ray diffraction (XRD) patterns of the precursor gels before the hydrothermal treatment. The XRD patterns of the precursor gels with molar ratios of Ag:Sr:Ti = 1:0:4 and 1:1:4 in Figure 1a,b show an amorphous pattern, while the XRD peaks identified as Sr(CH_3_COO)_2_·0.5H_2_O are found for the XRD patterns of the precursor gels with molar ratios of Ag:Sr:Ti = 1:2:4, 1:3:4 and 1:4:4 (Figure 1c,d,f)). Since there is no peak identified as CH_3_COOAg for these precursor gels regardless of the Sr molar fractions, Ag^+^ ions are assumed to be preferentially incorporated into amorphous titanium oxide gel networks. In the case of the lower Sr molar fractions, Sr^2+^ ions are completely incorporated into the amorphous gel networks with Ag^+^ ions. However, for the higher Sr molar fractions (molar ratios of Ag:Sr:Ti = 1:3:4 and 1:4:4), excess Sr(CH_3_COO)_2_·0.5H_2_O crystals were precipitated. On the other hand, XRD patterns of the series of precursor gels with the various Ag molar fractions (the molar ratio of Sr:Ti was fixed to 1:1) are shown in Figure 1e–h. All of the XRD peaks are identified as Sr(CH_3_COO)_2_·0.5H_2_O for the precursor gels with molar ratios of Ag:Sr:Ti = 0:4:4, 1:4:4 and 2:4:4 (Figure 1e–g). In contrast, other than the Sr(CH_3_COO)_2_·0.5H_2_O peaks, the peaks identified as CH_3_COOAg are found for the precursor gel with a molar ratio of Ag:Sr:Ti = 4:4:4 (Figure 1h). Therefore, Ag^+^ ions are completely incorporated into the gel networks for the precursor gels with the lower Ag molar fractions (molar ratios of Ag:Sr:Ti = 1:4:4 and 2:4:4). For these precursor gels, a certain amount of Ag^+^ and Sr^2+^ ions are incorporated into the amorphous titanium oxide gel networks, and excesses of Sr(CH_3_COO)_2_·0.5H_2_O and CH_3_COOAg are precipitated.

### 2.2. Synthesis of Hybrid Particles from Precursor Gels with Various Sr Molar Fractions

**Figure 2:**
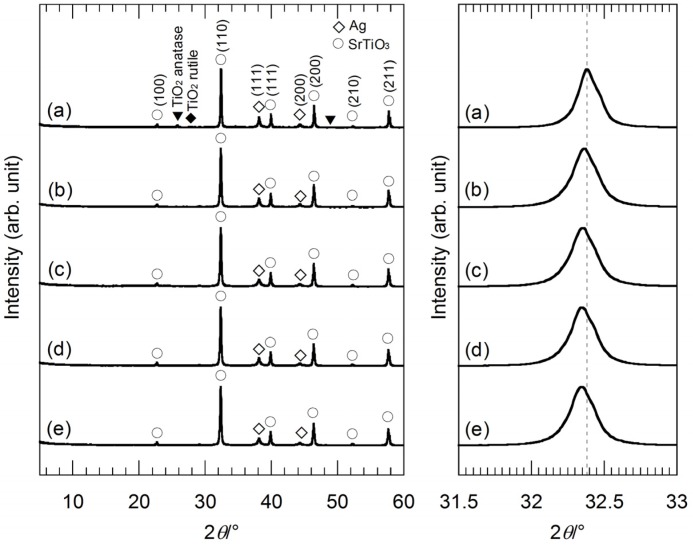
(**Left**) XRD patterns of the hybrid particles synthesized from the precursor gels with molar ratios of (a) Ag:Sr:Ti = 1:0:4; (b) 1:1:4; (c) 1:2:4; (d) 1:3:4 and (e) 1:4:4 by the hydrothermal treatment at 230 °C for 6 h in Sr(OH)_2_ aqueous solutions. (**Right**) XRD peaks of the hybrid particles indexed as (110) of SrTiO_3_ in the 2θ range of 31.5°–33.0°. The slight peak-shift can be clearly seen.

Figure 2 shows XRD patterns of the hybrid particles synthesized from the precursor gels with molar ratios of Ag:Sr:Ti = 1:0:4, 1:2:4, 1:3:4 and 1:4:4 by the hydrothermal treatment in strontium hydroxide (Sr(OH)_2_) aqueous solutions. The hydrothermally-synthesized sample powder from the precursor gel without Sr^2+^ ion (Ag:Sr:Ti = 1:0:4), which was reacted with Sr(OH)_2_, mainly consists of Ag and SrTiO_3_, though this sample contains impurities; anatase- and rutile-type TiO_2_ (Figure 2a). When the other precursor gels containing Sr^2+^ ions were used, we could obtain the Ag-SrTiO_3_ hybrid particles without impurity (Figure 2b–e), because the Sr source included in the precursor gels relatively increases and the Ti source included in the gels relatively decreases. In the hydrothermal reaction, the Sr^2+^ ions dissociated from the gel networks, and the excess Sr(CH_3_COO)_2_·0.5H_2_O could be also reacted with titanium oxide gels other than Sr^2+^ ions supplied by Sr(OH)_2_. Thus, the formation reaction of SrTiO_3_ is considered to be facilitated for the precursor gels with the higher Sr molar fractions. In Figure 2, the XRD peaks originating from Ag tend to broaden with a decrease in the Ag molar fraction in the precursor gels. This suggests that the size of Ag nanoparticles becomes smaller by using the gels with the lower Ag molar fractions, because of a decrease in the supply of the Ag source. A comparison of the XRD peaks indexed as (110) of SrTiO_3_ reveals a slight diffraction peak-shift. All of the XRD peaks originating from SrTiO_3_ of the hybrid particles synthesized from the gels containing Sr^2+^ ions (Figure 2b–e) slightly shift toward a lower 2θ angle as compared to the hybrid particles from the precursor gel without Sr^2+^ ions (Figure 2a). Ag^+^ ions can be doped into SrTiO_3_, because the Ag^+^ ion radius is comparable to the Sr^2+^ ion radius [20]. However, it is difficult to confirm the doping of Ag^+^ by the XRD measurement due to the similarity of the ion radii. For the case of BaTiO_3_ particles synthesized by the hydrothermal method, it was reported that an incorporation of hydroxyl ions in the crystal lattice enlarges the cell volume [21,22,23,24]. Therefore, we investigated the presence of the hydroxyl defects in the synthesized SrTiO_3_ crystals by a thermogravimetric analysis.

**Figure 3:**
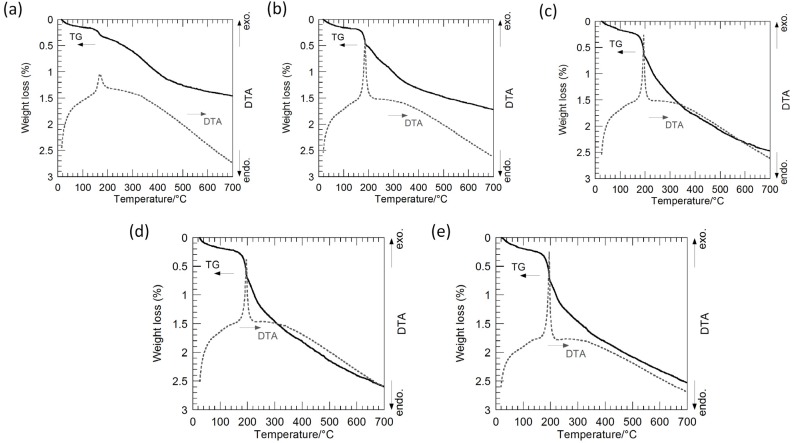
Thermogravimetry-differential thermal analysis (TG-DTA) curves of the Ag-SrTiO_3_ hybrid particles synthesized from the precursor gels with molar ratios of (**a**) Ag:Sr:Ti = 1:0:4; (**b**) 1:1:4; (**c**) 1:2:4; (**d**) 1:3:4; and (**e**) 1:4:4 by the hydrothermal treatment at 230 °C for 6 h in Sr(OH)_2_ aqueous solutions.

Figure 3 shows thermogravimetry-differential thermal analysis (TG-DTA) curves of the hybrid particles synthesized from the precursor gels with molar ratios of Ag:Sr:Ti = 1:0:4, 1:1:4, 1:2:4, 1:3:4 and 1:4:4 via the hydrothermal treatment at 230 °C for 6 h in Sr(OH)_2_ aqueous solutions. The weight loss is up to approximately 2.5%, and these hybrid particles are considered to contain a small amount of hydroxyl groups. The slight weight loss accompanied by the sharp exothermic peak is found at approximately 180 °C for all of the hybrid particles. The weight loss of the hybrid particles synthesized by the hydrothermal treatment in a range from 200 to 700 °C, which may be attributed to the hydroxyl ions incorporated in the SrTiO_3_ crystals, monotonically increases from 1.1% to 1.9% with the Sr molar fraction in the precursor gels. Accordingly, the shift of the SrTiO_3_ XRD peaks is attributed to the incorporation of hydroxyl groups in the SrTiO_3_ crystals.

**Figure 4:**
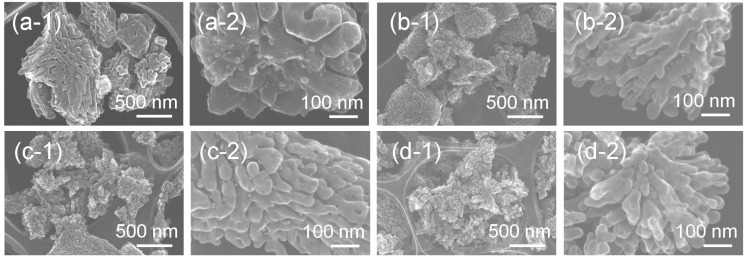
Secondary electron images of the Ag-SrTiO_3_ hybrid particles synthesized from the gels with a molar ratio of (**a**) Ag:Sr:Ti = 1:0:4, (**b**) 1:1:4, (**c**) 1:2:4 and (**d**) 1:3:4 by the hydrothermal treatment at 230 °C for 6 h in Sr(OH)_2_ aqueous solutions obtained by the STEM at (**a-1**–**d-1**) low and (**a-2**–**d-2**) high magnifications.

Secondary electron images, obtained by a scanning transmission electron microscope (STEM), of these hybrid particles synthesized from the gels with molar ratios of Ag:Sr:Ti = 1:0:4–1:3:4 via the hydrothermal treatment are shown in Figure 4. The synthesized Ag-SrTiO_3_ hybrid particles have a dendritic structure consisting of many branched nanoparticles. The particle size of the particles is sub-micro- to micro-meter order, and the particle size decreases with the Sr molar fraction in the precursor gels. The formation of the particles with similar hierarchical structures was reported for the other perovskite compounds. Maxim *et al.* [25] reported the hydrothermal synthesis of the dendritic BaTiO_3_ particles by using layered titanate as a precursor. Daniels *et al.* [26] reported dendritic La_0.5_Sm_0.5_CrO_3_ particles synthesized by the sol-gel-hydrothermal method. Other research groups reported the synthesis of single-crystal BaTiO_3_ [27], Ba_1−*x*_Sr*_x_*TiO_3_ [28] and SrTiO_3_ dendrites [29]. We have also reported dendritic Ag-BaTiO_3_ hybrid particles by using the gels derived by the sol-gel process [15]. Such dendritic growth is promoted under the non-equilibrium condition, in which the growth rate of crystals sufficiently exceeds the mass transport rate of ions for the crystal growth (diffusion-limited aggregation phenomena) [25,27,29,30,31,32]. In our present study, we consider that the formation mechanism of the dendritic hybrid nanoparticles is explained by an analogy to these dendritic perovskite particles. During hydrothermal treatment, the amorphous titanium oxide gels with Ag^+^ and Sr^2+^ ions are locally dissolved in basic solutions, and SrTiO_3_ nuclei immediately form by the reaction of the dissolved titanium oxide gels with Sr^2+^ ions in the reaction solution and/or in the dried gel itself. As the sol-gel-derived amorphous titanium oxide gels can be considered to act as highly reactive precursors, the nucleation rate of SrTiO_3_ is fast, and a large amount of nuclei is formed in the reaction solution [22]. The high surface tension anisotropy induced in the nuclei, owing to a large number of defects, gives rise to dendritic growth in the diffusion-limited aggregation phenomena [25]. On the other hand, the dissolution of the precursor gels is accompanied by a release of Ag^+^ ions. Thus, the local concentration of Ag^+^ ions around the growing SrTiO_3_ dendrites increases, and these Ag^+^ ions react with OH^−^ ions and immediately form nanocrystalline precipitates on the surface of SrTiO_3_ dendrites. Ag_2_O is expected to be formed in basic aqueous solutions at room temperature, but Ag_2_O is then reduced under the hydrothermal conditions [15]. As a result of competitive formation reactions of Ag and SrTiO_3_ around the precursor gels, Ag nanoparticle-loaded SrTiO_3_ hybrid nanoparticles can be obtained.

### 2.3. Synthesis of Hybrid Particles from Precursor Gels with Various Ag Molar Fractions

**Figure 5:**
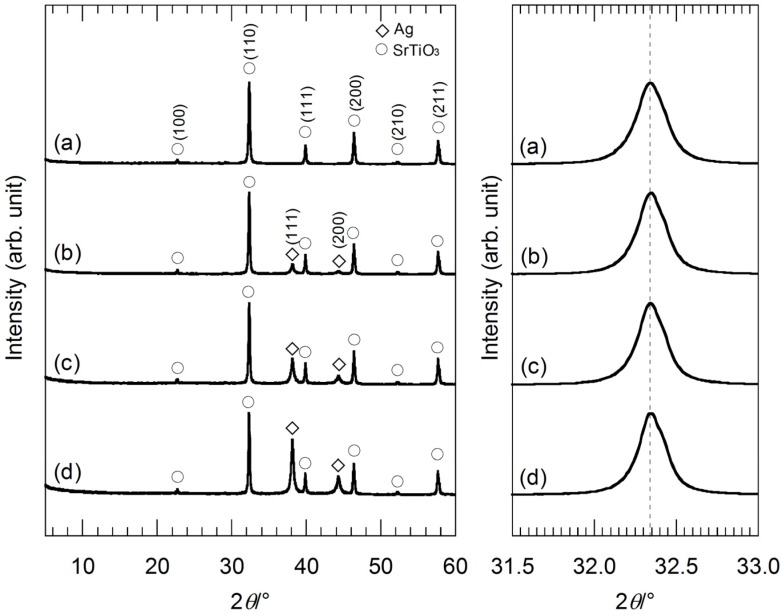
(**Left**) XRD patterns of the hybrid particles synthesized from the precursor gels with molar ratios of (a) Ag:Sr:Ti = 0:4:4; (b) 1:4:4; (c) 2:4:4; and (d) 4:4:4 by the hydrothermal treatment at 230 °C for 6 h in Sr(OH)_2_ aqueous solutions; (**Right**) XRD peaks of the hybrid particles indexed as (110) of SrTiO_3_ in the 2θ range of 31.5°–33.0°.

Figure 5 shows the XRD patterns of the hybrid particles synthesized from the precursor gels with molar ratios of Ag:Sr:Ti = 0:4:4, 1:4:4, 2:4:4 and 4:4:4 by the hydrothermal treatment at 230 °C for 6 h in Sr(OH)_2_ aqueous solutions. All of the XRD peaks are identified as Ag and SrTiO_3_, suggesting the formation of the Ag-SrTiO_3_ hybrid particles. The broadness of the XRD peaks originating from Ag is almost the same for all the hybrid particles, and thus, the crystalline size of the Ag nanoparticles is assumed to be similar. In Figure 5, the shift of the XRD peaks of the hybrid particles indexed as (110) of SrTiO_3_ in the 2θ range of 31.5°–33.0° is not found. The XRD peak-shift is related to the Sr molar fractions in the precursor gels.

**Figure 6:**
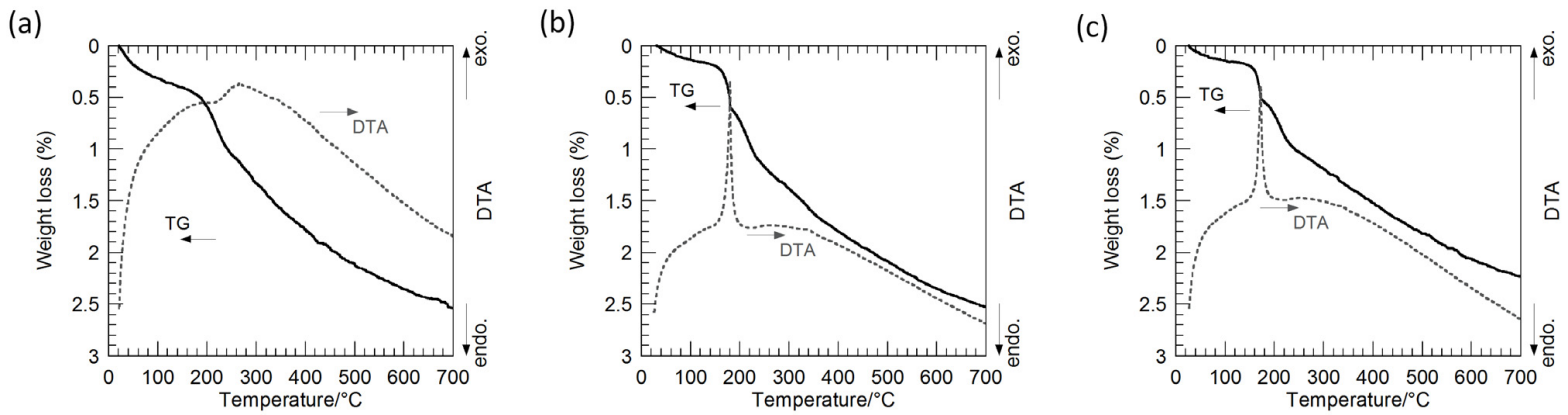
TG-DTA curves of the SrTiO_3_ and the Ag-SrTiO_3_ hybrid particles synthesized from the precursor gels with molar ratios of Ag:Sr:Ti = (**a**) 0:4:4; (**b**) 2:4:4; and (**c**) 4:4:4 by the hydrothermal treatment at 230 °C for 6 h in Sr(OH)_2_ aqueous solutions.

Figure 6 shows the TG-DTA curves of these hybrid particles synthesized from the precursor gels with the various Ag molar fractions, and a weight loss of 2.2%–2.6% up to 700 °C is found for all the hybrid particles. The sharp exothermic peaks appear at around 180 °C in the DTA curves of the Ag-SrTiO_3_ hybrid particles, while such exothermic peaks cannot be found for the SrTiO_3_ particles without Ag. Accordingly, it is suggested that the precursor gels were almost completely converted into Ag and SrTiO_3_ by the hydrothermal treatment at 230 °C for 6 h in Sr(OH)_2_ solutions, but a small amount of the hydroxyl groups might be present. The slightly large weight loss of around 1.7%–2.0% in the range from 200 to 700 °C, which may be attributed to the hydroxyl ions incorporated in the SrTiO_3_ crystals, is found for these SrTiO_3_ and Ag-SrTiO_3_ hybrid particles synthesized from the gels with molar ratios of Ag:Sr:Ti = 0:4:4, 1:4:4, 2:4:4 and 4:4:4 at 230 °C for 6 h in Sr(OH)_2_ aqueous solutions. It is considered that these hydroxyl defects induce the enlargement of the SrTiO_3_ lattices and result in the XRD peak-shift toward a lower 2θ angle (Figure 5a–d) as compared to the hybrid particles from the precursor gel without Sr^2+^ ions (Figure 2a).

**Figure 7:**
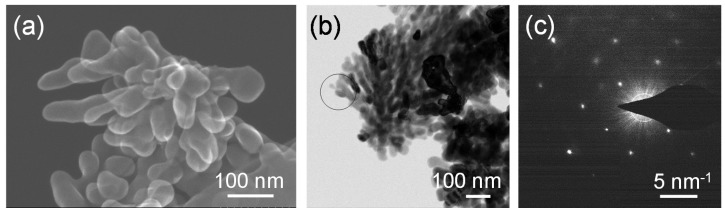
(**a**) A secondary electron image obtained by the STEM; and (**b**) Field-emission transmission electron microscope (FE-TEM) images of the SrTiO_3_ particles synthesized from the gel with a molar ratio of Ag:Sr:Ti = 0:4:4 by the hydrothermal treatment at 230 °C for 6 h in Sr(OH)_2_ aqueous solutions; (**c**) electron diffraction pattern of the SrTiO_3_ particles synthesized from the gels without Ag (the molar ratio of Ag:Sr:Ti = 0:4:4) obtained by the selected area shown in (**b**).

Figure 7a,b shows a secondary electron image obtained by the STEM and field-emission transmission electron microscope (FE-TEM) images of the SrTiO_3_ particles synthesized from the gel with the molar ratio of Ag:Sr:Ti = 0:4:4 at 230 °C for 6 h in Sr(OH)_2_ solutions, respectively. It can be clearly seen that dendritic nanoparticles with a size of a few hundreds of nanometers were formed. The selected-area electron diffraction (SAED) analysis was performed for the dendritic SrTiO_3_ particle shown in Figure 7b, and the spot pattern was obtained for the corresponding area (Figure 7c). A dendritic SrTiO_3_ particle is not a single crystal, but a part of the branches may have a single-crystalline nature.

**Figure 8:**
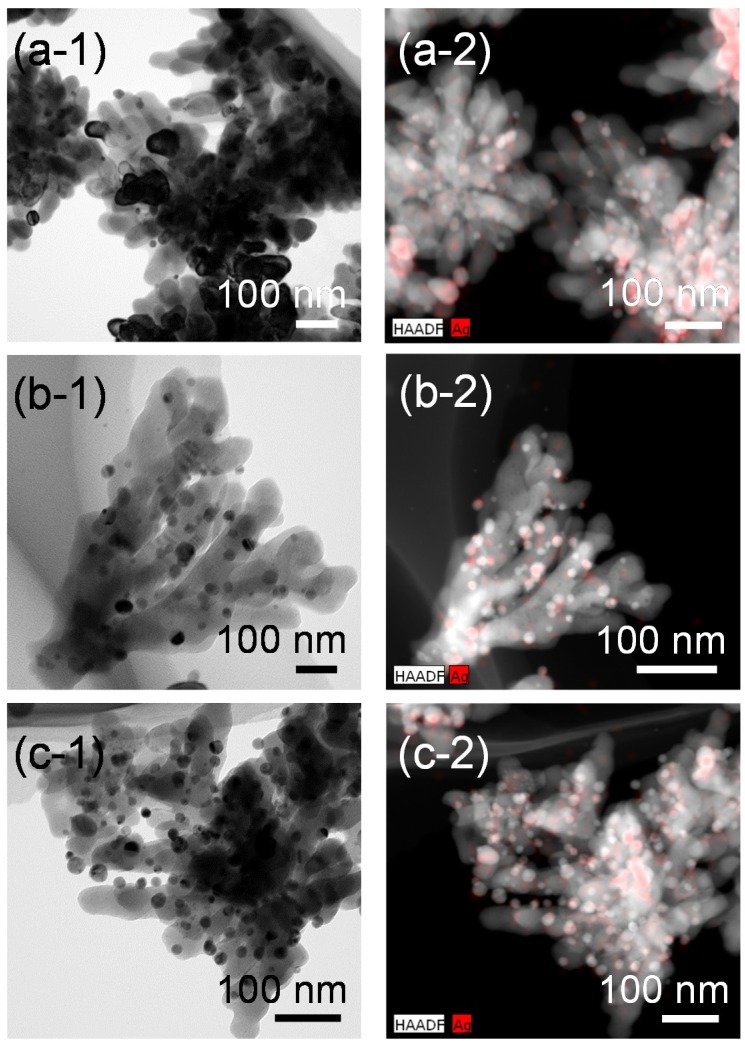
FE-TEM images of the Ag-SrTiO_3_ hybrid particles synthesized from the gels with molar ratios of (**a-1**) Ag:Sr:Ti = 1:4:4; (**b-1**) 2:4:4 and (**c-1**) 4:4:4 by the hydrothermal treatment at 230 °C for 6 h in Sr(OH)_2_ aqueous solutions. (**a-2**–**c-2**) Corresponding high-angle annular dark field (HAADF)-STEM images with STEM-energy dispersive X-ray spectroscopy (EDX) mapping of the hybrid particles shown in (**a-1**–**c-1**), respectively. Red-colored regions correspond to the Ag nanoparticles.

Figure 8 shows FE-TEM images and the corresponding high-angle annular dark field (HAADF)-STEM images with STEM-energy dispersive X-ray spectroscopy (EDX) mapping of the Ag-SrTiO_3_ hybrid particles synthesized from the gels with the various Ag fractions by the hydrothermal treatment at 230 °C for 6 h in Sr(OH)_2_ solutions. We can see the Ag nanoparticles are loaded on the SrTiO_3_ dendritic nanoparticles, which may have a high specific surface area. There is a wide size distribution of the adsorbed Ag nanoparticles, but the Ag nanoparticles with a size of a few tens of nanometers are distributed without severe agglomeration in these observation areas. By using the sol-gel-derived amorphous titanium oxide gels incorporated with the metal source as the precursors, we could obtain the Ag nanoparticle-loaded SrTiO_3_ dendritic nanoparticles by the hydrothermal treatment at low temperature. These Ag-SrTiO_3_ hybrid nanoparticles are expected to be applied to the high-capacitance Ag/SrTiO_3_ composite materials or the photocatalysts.

## 3. Experimental Section

The precursor gels were prepared by the conventional sol-gel process. Twelve point five millimolar per decimeter cubed of CH_3_COOAg (Wako Pure Chemical, 97.0%, Osaka, Japan) and 0, 12.5, 25, 37.5 and 50 mmol/dm^3^ Sr(CH_3_COO)_2_·0.5 H_2_O (Wako Pure Chemical, 99.0%) were completely dissolved in 15 mL of deionized water at 80 °C. On the other hand, 50 mmol/dm^3^ Ti[(CH_3_)_2_CHO]_4_ (Wako Pure Chemical, 95.0%) and 100 mmol/dm^3^ acetyl acetone (CH_3_COCH_2_COCH_3_, Wako Pure Chemical, 99.0%) were mixed with 15 mL of ethanol. Then, these aqueous and ethanolic solutions were completely mixed, and homogeneous solutions with various Sr molar fractions (molar ratios of Ag:Sr:Ti = 1:0:4, 1:1:4, 1:2:4, 1:3:4 and 1:4:4) were prepared.

We carried out another set of experiments in which we changed the concentration of CH_3_COOAg for the aqueous solutions; 0, 12.5, 25, 37.5 and 50 mmol/dm^3^ CH_3_COOAg and 50 mmol/dm^3^ Sr(CH_3_COO)_2_·0.5H_2_O were completely dissolved in 15 mL of deionized water at 80 °C. These aqueous solutions were mixed with 15 mL of 50 mmol/dm^3^ Ti[(CH_3_)_2_CHO]_4_ and 100 mmol/dm^3^ CH_3_COCH_2_COCH_3_ ethanolic solutions and were turned into homogeneous solutions with various Ag molar fractions (molar ratios of Ag:Sr:Ti = 0:4:4, 2:4:4 and 4:4:4). After these mixed solutions were dried at 120 °C, these gels were ground into powders, and the precursor gels were obtained.

One hundred milligrams of the precursor gels were added to 10 mL of the strontium hydroxide octahydrate (Sr(OH)_2_·8H_2_O, Wako Pure Chemical, 90.0%) aqueous solution with a concentration of 75 mmol/dm^3^. Then, the hydrothermal treatment was performed at 230 °C for 6 h with a heating rate of 5 °C/min in an autoclave. The resultant powders were washed with 3 vol% of formic acid aqueous solution and were dried at 80 °C.

The crystal structure of the samples was identified by XRD measurement using Cu Kα radiation (Ultima IV, Rigaku Co., Tokyo, Japan). The thermal decomposition behavior of the precursor gel was examined by TG-DTA (DTG-60, Shimadzu, Kyoto, Japan) preformed with a heating rate of 10 °C/min in air. Microstructures of the sample powders were observed by STEM (HD-2300C, Hitachi High-Technologies, Hitachi, Japan) equipped with a secondary electron detector and a filed-emission transmission electron microscope (FE-TEM; Osiris, FEI, Hillsboro, OR, USA) equipped with EDX.

## 4. Conclusions

The Ag nanoparticle-loaded SrTiO_3_ nanoparticles were successfully synthesized from the titanium oxide gels incorporated with Ag^+^ ions by the sol-gel-hydrothermal method at 230 °C for 6 h in Sr(OH)_2_ aqueous solutions. According to the TG analysis, these hybrid particles contain a small amount of hydroxyl defects. The morphology of the SrTiO_3_ nanoparticles is dendritic in the presence and absence of Ag^+^ ions. The amorphous titanium oxide gels with Ag^+^ and Sr^2+^ ions, which act as the high reactive precursor, give rise to high nucleation and growth rates under the hydrothermal conditions, and the resultant diffusion-limited aggregation phenomena facilitate the dendritic growth of SrTiO_3_. From the FE-TEM observation of these Ag-SrTiO_3_ hybrid nanoparticles, the Ag nanoparticles with a size of a few tens of nanometers are distributed without severe agglomeration, owing to the competitive formation reactions of Ag and SrTiO_3_.
